# The Use of a Recombinant Canarypox-Based Equine Influenza Vaccine during the 2007 Australian Outbreak: A Systematic Review and Summary

**DOI:** 10.3390/pathogens5020042

**Published:** 2016-06-10

**Authors:** Romain Paillot, Charles M. El-Hage

**Affiliations:** 1 Animal Health Trust, Lanwades Park, Kentford Newmarket, CB8 7UU Suffolk, UK; 2 The Centre for Equine Infectious Diseases, Veterinary Pre-Clinical Centre, University of Melbourne, VIC 3010, Australia; cmeh@unimelb.edu.au

**Keywords:** equine influenza, horse, vaccination, canarypox-based vaccine, DIVA

## Abstract

In 2007, Australia experienced the most extensive equine influenza outbreak observed in recent years. Extraordinary measures were rapidly implemented in order to control and prevent the spread of this highly contagious disease. The control strategy involved stringent movement restriction and disease surveillance, seconded by emergency post-outbreak vaccination strategies. Sixteen months after the first case and 12 months following the last reported case, Australia regained its equine influenza-free OIE status. This systematic review reports and summarises information relating to the implementation of emergency vaccination during the 2007 Australian equine influenza outbreak, including the choice of vaccine and implementation strategies.

## 1. The Outbreak

Equine influenza (EI) is a major respiratory disease of the horse caused by the highly contagious equine influenza virus (EIV). Together with Iceland and New Zealand, Australia had remained free from EI until August 2007 when the first cases of EI were diagnosed in New South Wales (NSW).

This outbreak originated from the importation of one or more sub-clinically infected horses from Japan, which was experiencing an EI outbreak at the time [1]. These horses had responded poorly to recent vaccination or were infected during the immunity gap, a period of low protective immunity that may occur in some vaccinated horses between the primary vaccination course and the first boost immunization [2,3,4]. Vaccination had not been permitted in Australia, which had remained free of EI, hence the horse population was naïve to EIV. The breach of quarantine at the Eastern Creek Animal Quarantine Station and the subsequent virus escape led to a significant outbreak, with over 76,000 horses infected with EIV detected on more than 10,600 properties [3].

The EIV strain A/equine/Sydney/2888-8/07 (H3N8), representative of the Australian EI outbreak, was classified as a member of the Florida sublineage clade 1 (FC1), typical of viruses isolated in North America at the time and closely related to A/equine/Ibaraki/07, the representative strain from the Japanese 2007 EI outbreak [5]. The Australian EI outbreak was first detected on 24th August, peaked on 1st October and lasted 5 months, with the last known case reported in December 2007 [6]. Around 8% of the susceptible Australian horse population was infected with EIV [7].

## 2. Control Strategies (Movement Restriction + Vaccination Policy)

Once EI was detected in the general population, the spread was contained within those areas that were infected prior to discovery. The propagation rate inside the infected areas was reported in some cases to be around 1 to 1.5 km/day, in the absence of horse-to-horse or human-to-horse contact [8]. Following the initial dissemination of EIV that occurred at several horse competitions (Maitland, Narrabi (NSW) and Warwick (Queensland, QLD)), an immediate 72-h nationwide “horse standstill” was imposed on 25th of August [3].

Within 3 weeks of the start of the outbreak, a zoning system based on local Government boundaries was implemented in NSW, including purple zones (special restricted areas considered infected but with no internal restriction for movement), red zones (restricted area, no movements and/or events, initially 10 km around infected premises (IP)), amber zones (controlled area, 50 km around red zone) and green zones (protected, no restrictions) [8]. Horse movement was banned between premises in both Red and Amber zones. Queensland was classified in three zones (red, amber and green) [9].

A surveillance programme was put in place in affected states, which covered over 44,000 horses in 9700 premises (NSW and QLD). Equine influenza surveillance was also undertaken in those states and territories of Australia with no confirmed EI cases [6]. All these measures contributed to limiting the spread of EIV through horse-to-horse contact and airborne dissemination (estimated around 1.27 to 2.38 km in peri-urban and rural areas, respectively [8,10]).

An emergency vaccination programme was initiated in late September some 4 weeks after the outbreak was declared, and involved between 130,000 to 170,000 horses [10,11,12]. The vaccination strategy (reactive and prophylactic) included ring vaccination around infected areas (10 km wide vaccination buffer zones), predictive vaccination (targeted horse populations posing strategic or economic risk to contribute to spread of the disease, such as Thoroughbred racehorses, competition horses, police horses, *etc.*) and blanket vaccination (to maximise immunity in specific area) [9,11]. Prophylactic vaccination was also used to ensure that the Victorian Spring Racing Carnival featuring the Melbourne Cup would not be jeopardised. Movement of vaccinated horses from infected to non-infected areas was eventually authorised under a strict process of pre- and post-movement isolation and testing (two qRT-PCR tests detecting type A influenza viruses required during the pre-movement isolation and the possibility to undertake a further qRT-PCR test at the post-movement isolation if necessary) [13,14].

Equine influenza was detected in and contained to approximately 3.5% of the Australian land mass [7]. Provisional freedom from EI was declared on 14 March 2008 and full freedom on 30 June 2008. In December 2008, twelve months after the last reported case, Australia regained its EI-free status [15] from the OIE.

## 3. The Choice of Vaccine and Vaccination Implementation

Equine influenza vaccines that were commercially available at the time of the outbreak were reviewed by the Consultative Committee for Emergency Animal Disease (CCEAD). A live recombinant (canarypox) vectored vaccine (ProteqFlu^®^; Merial) was selected [16,17]. The ProteqFlu vaccine used in Australia contained two recombinant canarypox viruses expressing haemagglutinin (HA) from the EIV strains A/equine/Newmarket/2/93 (H3N8, European lineage) and A/equine/Kentucky/94 (H3N8, American lineage). Like all commercialised EI vaccines at the time, the canarypox-vectored EI vaccine did not fully meet the OIE expert surveillance panel recommendations on EI vaccine strain composition (*i.e*., inclusion of an A/equine/South Africa/4/03-like virus (American lineage) and an A/equine/Newmarket/2/93-like virus (European lineage) [18]). However, the successful use of this vaccine during the 2003 South African EI outbreak [19] and the possibility to use an EIV nucleoprotein (NP)-specific ELISA to differentiate infected from vaccinated animals (DIVA), were important elements in favour of the canarypox-vectored vaccine for selection by the CCEAD [11].

The first shipment of the recombinant canarypox EI vaccine arrived in Australia on 27th September 2007 (20,000 doses of both ProteqFlu and ProteqFlu TE, which contains tetanus toxoid) [11]. In total, more than 321,500 doses of vaccines were used, with at least 100,000 horses receiving the primary immunisation programme (*i.e*., two doses) [7,11].

### 3.1. Vaccination Buffer Zone

**Figure 1:**
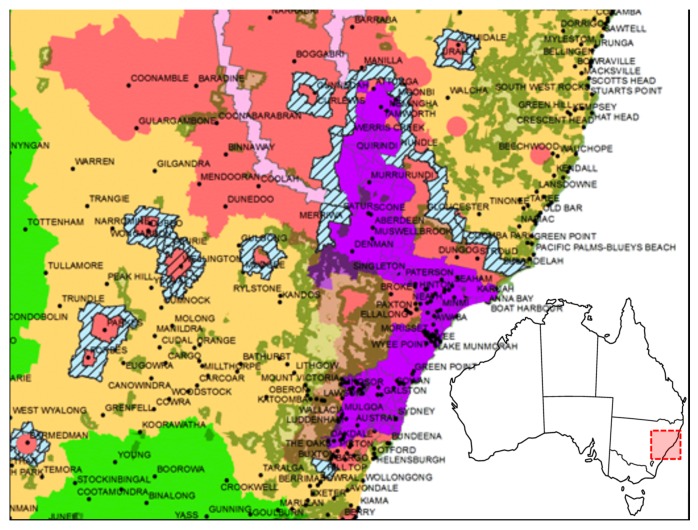
Map of zoned areas in NSW indicating equine influenza (EI) movement restrictions. Blue lined zones around red areas indicate buffer zones of vaccinated horses (image courtesy of NSW Department of Primary Industries (DPI) Equine Influenza Symposium).

Vaccination rings (10 km wide or wider), also called vaccination buffer zones (VBZ), were implemented within or around infected areas (often classified as suppressive or protective vaccinations, respectively) [11]. Later, modelling of vaccine efficacy based on the epidemiological analysis of the outbreaks indicated that suppressive vaccination strategy (1 to 3 km ring vaccination) around infected areas gave the most effective results in terms of new infections reduction (−64%) and infected area (−9%) [20,21]. In QLD, a layered system of outer and inner VBZs (25–35 km apart) was put in place around known infected areas. Further vaccination extensions between the inner and outer vaccination zones were developed in order to compartmentalise QLD [9,22]. Ring vaccination was also used to prevent EIV transmission between foci of infection and wild horse populations in NSW (5,000 to 8,000 animals) in order to avoid the establishment of an EIV reservoir. No transmission to wild/feral horses was detected [9,23]. Vaccination buffer zones in NSW are illustrated in Figure 1. Overall, 80% to 90% of horses in QLD and NSW VBZs were vaccinated by the end of November 2007 [11]. The rate of EIV transmission/infection and the subsequent morbidity decreased with increased rate of vaccination. However, it is important to note that presence of vaccinated horses sub-clinically infected may have complicated clinical surveillance and subsequent localisation and delineation of the outbreak [24]. Overall, it is generally accepted that vaccination contributed to limit further spread of EIV infection [11].

### 3.2. Schedule of Vaccination

The recommended vaccination schedule for the recombinant canarypox EI vaccine is composed of a primary course (first immunisation (V1) from 5 to 6 months of age with a second immunisation (V2) 4–6 weeks later) and revaccination 5 months later (V3). Field results from the 2007 Australian outbreak report that frequency of infection, severity and duration of clinical signs of disease, and duration of virus shedding were significantly reduced in vaccinated horses when exposed only a few days after the first immunisation with the canarypox-based EI vaccine, when compared to unvaccinated horse populations. These data support a rapid onset of immunity after only one immunisation [25,26]. These observations confirmed previously reported early onset of immunity with stimulation of significant protective immunity 14 days after only one immunisation with the canarypox-based EI vaccine [27].

An accelerated schedule of vaccination (reduced to 14 days between the first and second immunisation) was also tested during the 2007 Australian outbreak. This accelerated regimen was implemented in order to improve vaccine administration flexibility, and reduce the length of time prior to stimulation of full protective immunity (usually observed 2 weeks after the second immunisation), which could prove extremely useful in an emergency situation, such as in the Australian outbreak. Protective levels (>85 mm^2^ and >154 mm^2^ for clinical and virological protection, respectively) of SRH (single radial haemolysis) antibodies were measured in such immunisation conditions and up to 4 months after V3 [28,29] (study co-funded by Merial Australia Pty Ltd, University of Melbourne and Victoria’s DPI). This accelerated schedule of vaccination was applied in several Australian states, including NSW and Victoria [30]. However, it was not possible to evaluate the potential impact of the accelerated schedule on the long-term duration of immunity (e.g., up to annual boost immunisation).

Equine influenza vaccination is usually recommended in foal of 5–6 months of age [31]. Foals as young as 4 months of age could be immunised with the recombinant canarypox EI vaccine [32]. However, at 4-months, immunisation should be followed by the full primary vaccination programme (*i.e*., V1 at 5–6 months of age followed by V2, 4–6 weeks later). As part of the vaccination strategy, the decision was taken to immunise all foals regardless of age, in order to increase vaccine coverage in the VBZs [11].

### 3.3. Serological Assays/Differentiating Infected from Vaccinated Animals (DIVA)

**Figure 2:**
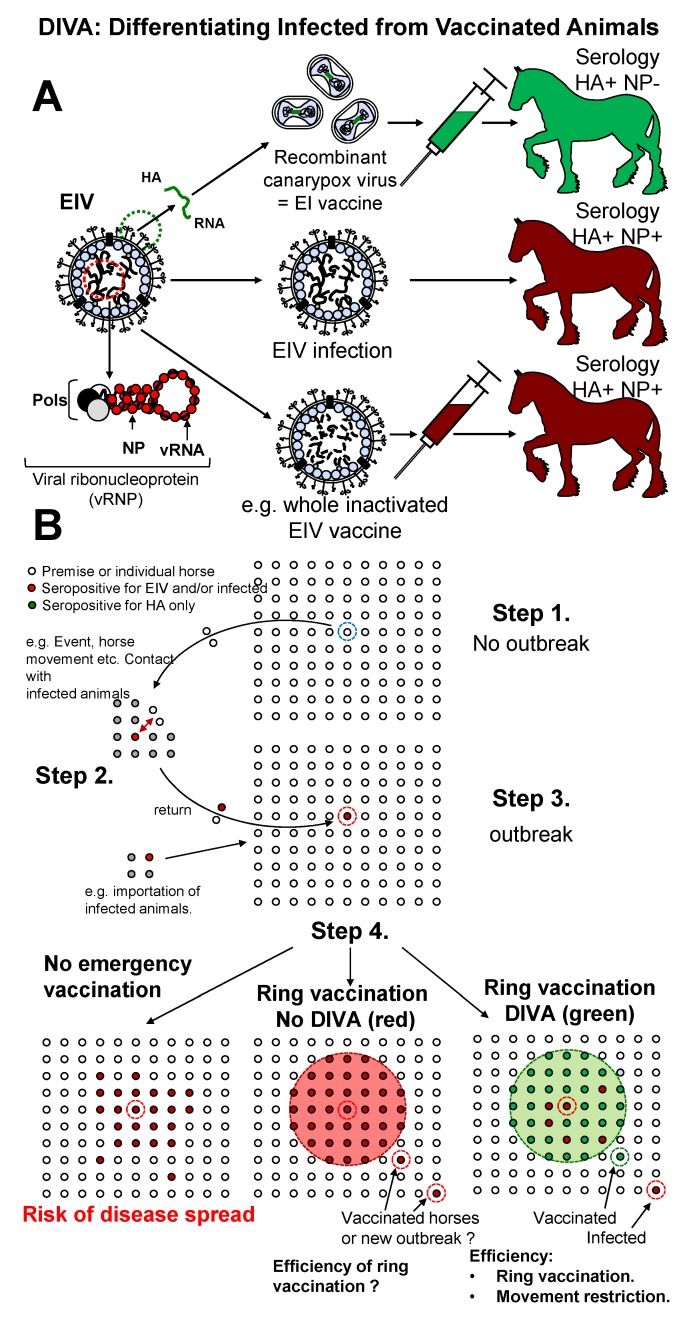
Differentiating Infected from Vaccinated Animals (DIVA). (**A**) Horses immunized with the recombinant canarypox-based EI vaccine seroconvert to haemagglutinin (HA) only (green). Horses infected with equine influenza virus (EIV) or vaccinated with a whole inactivated EI vaccine seroconvert to all EIV antigen (red), including the EIV nucleoprotein (NP) forming the viral ribonucleoprotein complex; (**B**) Importance of DIVA for emergency vaccination in the face of an EI outbreak: in an area initially free of EI (Step 1), a case of EI is detected (red spot, Step 3), following horse movement and importation of EIV (as an example, Step 2). Prevention measures are implemented (Step 4). In absence of EI vaccination, control of the disease relies entirely on movement restriction, surveillance and is influenced by the horse population density. EI is likely to spread. Ring vaccination could be implemented to support those control measures. In absence of DIVA capacity (red), the origin of seropositive horses found outside the vaccination area could not be determined (e.g., vaccinated horses outside the vaccination area or presence of the virus in the few days prior to serological analyses). The use of a DIVA EI vaccine allows determination of EIV progression inside and outside the vaccination area (*i.e*., ring vaccination efficacy) and evaluation of compliance with prevention measures such as movement restriction.

In contrast to other EI commercially available vaccines in 2007, the canarypox-based EI vaccine encodes the EIV HA gene only. Horses immunised with this specific EI vaccine only seroconverted against the EIV HA protein. An H3-subtype-specific haemagglutination inhibition (HI) assay [33] was used to confirm seroconvertion [13,34]. The HI antigen was prepared from an early Australian EIV isolate [13]. The SRH assay [33] is a serological method of choice to measure antibody response after vaccination due to a greater reproducibility than the HI test and well described correlation with levels of clinical and virological protection [35,36]. However, this method is more labour intensive and requires a longer incubation period (*i.e*., 20–24 h) when compared to the HI test, hence could be considered less advantageous when used for diagnostic purposes. To our knowledge, the SRH assay was not used in Australia during the outbreak. Horses infected with EIV mount an antibody response against most EIV structural proteins, including NP. A type A influenza group reactive blocking ELISA that targets a recombinant influenza viruses NP protein (NP-ELISA) was also used during the outbreak [13]. The NP-ELISA detects antibody response against the EIV NP, and was able to discriminate horses immunised with the recombinant canarypox EI vaccine from infected horses [34,37]. Differentiation of infected animals (HI+ and NP-ELISA+) from vaccinates (HI+ and NP-ELISA-) was of great importance for the Australian’s outbreak management and the HI test and NP-ELISA were successfully used as DIVA assays in Australia (Figure 2).

### 3.4. Adverse Reactions to Vaccination

Most adverse events observed after vaccination were classified as mild or minor. A transient (1–5 days) and limited (<5 cm) swelling at the site of injection was reported in up to 25% of Australian horses vaccinated with the recombinant canarypoxvirus EI vaccine [11,38]. Transient lethargy (<5% of horses), stiffness and swelling in the limbs (<1%). A single case of fibrosarcoma was reported at the site of administration following the second recombinant canarypox-based EI vaccination [39]. No deaths were linked to vaccination.

## 4. Evaluation of EI Vaccines against the Representative Australian EIV Strain

Several EI vaccines commercialised in the Europe Union (EU) at the time of the Australian outbreak were subsequently tested for efficacy in a controlled experimental vaccination/infection model against the representative Australian EIV strain A/equine/Sydney/2888-8/07 (H3N8). This study included the subunit ISCOM-based EI vaccine (EquipF) [40,41], a whole inactivated EI vaccine (Duvaxyn IE) [42] and the recombinant canarypox-based EI vaccine used in the field situation in Australia [41] ([40,41] sponsored by the Horserace Betting Levy Board, [42] sponsored by Fort Dodge Animal Health). These studies confirmed that emergency use of most EI vaccines commercially available in the EU in 2007/2008 would have provided statistically significant protection against the Australian FC1 EIV strain. This provided some re-assurance in the case of possible importation and circulation of an Australian-like EIV in the EU. Ponies vaccinated with the canarypox-based EI vaccine were significantly protected when experimentally infected with A/equine/Sydney/2888-8/07, with a marked reduction in clinical signs of disease (including coughing) and amount/duration of EIV shedding. These results support field clinical observations in a group of Australian racehorses naturally infected shortly after vaccination [26]. Vaccinated horses displayed no or reduced coughing episodes (37.6% affected, one cough per 20 min) when compared with non-vaccinated horses (100% affected, mostly >1 cough per 20 min). Reduced coughing and virus shedding is likely to have reduced the risk of EIV airborne transmission inside the VBZs.

## 5. Impact and Costs to the Industry

The overall cost of the 2007 EI outbreak to the Australian economy is difficult to estimate. Horse industries in NSW and QLD were most affected. The direct cost to owners was reported to reach A$100 million [43], with an average loss of A$1,000 per week for affected horse owners, A$3,000 per week for businesses [44] and over A$263 million in government assistance to the horse industry [7,43]. Surveys conducted in 2008 reported an estimated loss of A$381 to A$522 million by horse breeders, recreational owners and riding club members [7,44]. Total cost was estimated to reach A$1 billion. Two hundred and sixty one Standardbred race meetings were cancelled (164 in NSW and 82 in QLD) [7]. Specific biosecurity measures were put in place around Sydney to temporarily maintain racing events for several weeks to minimise the impact of the outbreak [30]. Racing NSW and the Thoroughbred breeding industry vaccinated more than 12,000 Thoroughbreds in order to achieve this continuity [30]. Economic losses within red and amber zones were considered to be substantial due to movement restrictions [44]. The Australian equine Olympic team selection was also affected as several qualifying events for the 2008 Beijing Olympic Games were cancelled or disrupted [44].

## 6. Consequences and Future Vaccination Implementation

An EI vaccine study was commissioned by Racing Victoria to evaluate different diagnostic methods and tools in order to optimise quarantine procedures. Duration of EIV shedding was measured in vaccinated horses (11 weeks post V2 with a sub-unit ISCOM based EI vaccine) after experimental infection with EIV (Florida Clade 2 strain A/equine/Richmond/1/07). Results indicated that vaccinated horses could transmit EIV to naïve horses for several days (the period of transmission is dependent on numerous factors, including time since last vaccination, the antigenic relationship between EI vaccine strains and circulating EIV strain). Nasopharyngeal swabbing and qRT-PCR were the best options to detect EIV shedding in vaccinated horses (when compared to nasal swabs for sampling and to EIV ELISA, titration in embryonated hens’ eggs and rapid serological test for diagnostic assays) [45].

Since 2007, the EI vaccination requirements for horse importation to Australia has been modified, with EI vaccination between 2–6 months prior to pre-exportation quarantine (PEQ) and with the recommendation to use an EI vaccine containing representative or currently circulating EIV strains. Testing for EIV in nasopharyngeal swab samples has been extended to PEQ as well as post-arrival quarantine (PAQ) [4]. Horses exported from Australia receive a whole inactivated EI vaccine. It was recommended to conduct export-related vaccination and nasal/nasopharyngeal swabs for EI testing at different times, to limit the risk of contamination and accidental false positive results in swab samples tested by qRT-PCR [46,47].

The importance and role played by emergency vaccination in Australia was debated, as the number of new infected cases was already decreasing at the time of vaccination, and natural protective immunity was increasing [9,10,48]. However models of the outbreak revealed that vaccine implementation; alongside biosecurity measures and movement restriction, were effective in the eradication of EI in Australia [20,21]. New Zealand (NZ) has never experience an EI outbreak. Different vaccination strategies (suppressive, protective and targeted) to control a potential EI outbreak in NZ have been recently evaluated. Simulation modelling results indicate that a rapid implementation of suppressive vaccination (*i.e*., 3 km radius around infected premises) would be effective to control the disease in NZ equine population, in association with movement restriction [49].

Early EI vaccination is now considered to be a strategic and key control measure in case of future EI outbreaks in Australia [50]. The EI vaccination strategy defined by the 2011 AUSVETPLAN involves:

- ring vaccination around infected areas (containment),
- predictive vaccination for high risk enterprises and densely populated areas,
- blanket vaccination in specific and infected areas,
- preventive vaccination in specific area/population to facilitate business continuity [51].

## 7. Materials and Methods

This review is written following the PRISMA statement [52]. The protocol, which is described here, was not registered with PRISMA.

### 7.1. Search Strategy (Figure 3)

In April 2016, a systematic literature search was conducted on equine influenza vaccination in horses during the 2007 Australian EI outbreak. Electronic databases were searched using MEDLINE (Pubmed with date limitation from 2007 to present) and Scopus (2007 to present). The search terms specified were ‘equine influenza vaccination Australia’ or ‘equine influenza vaccine Australia’ The language of publication is mostly English. Publication date ranged from 2007 to May 2016.

### 7.2. Publication Selection

**Figure 3:**
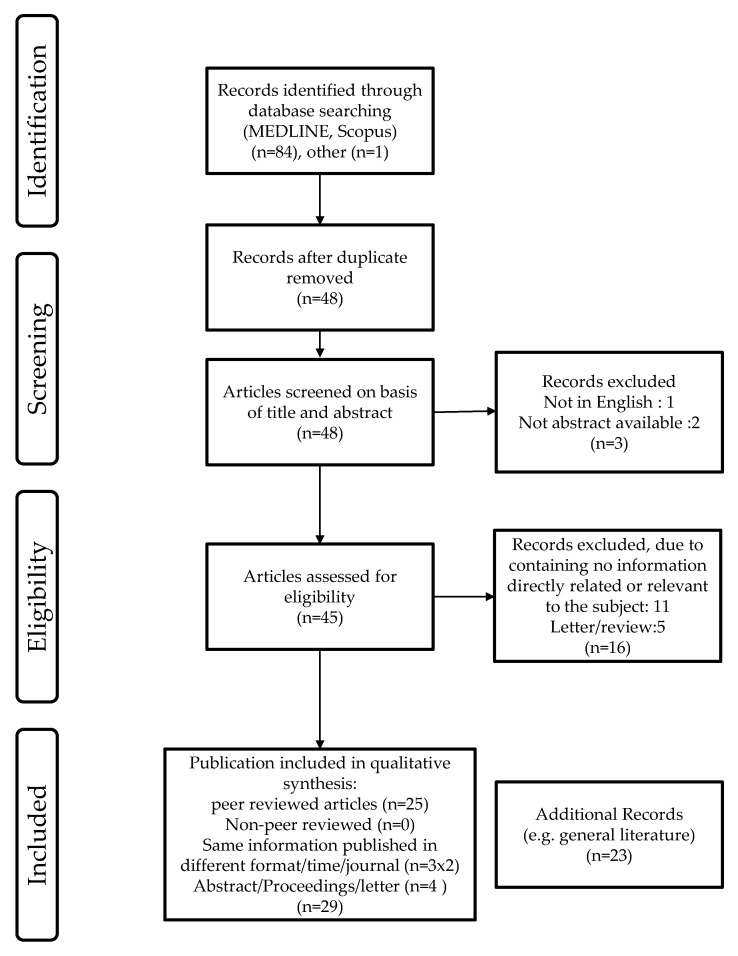
Systematic review process.

Publications were screened on title and abstracts. Those describing the field and/or experimental use of equine influenza vaccines in relation to the 2007 Australian EI outbreak were included. Publication date restriction was imposed (2007 to present) but no publication status restriction was imposed. Conference proceedings, manufacturers’ reports, patent and round-table discussions were also considered. Publications not in the English language were considered provided an abstract was available. Records were rejected if they were duplicates of data available as published articles. Studies published both in abstract and full paper forms were only evaluated in their full paper format. Duplicates are indicated in the review flow chart (Figure 3).

### 7.3. Data Collection Process and Items

Each selected publication was entered into a reference manager system (Endnote X2). The selected publications were ordered by primary authors to avoid duplication. Information was extracted from each included publication and reported in the review.

### 7.4. Risk of Bias in Individual Studies

When known, study sponsors were indicated in text to declare potential conflicts of interest.

## 8. Conclusions

Equine influenza vaccination is recognized as one of the most efficient methods to prevent, control and minimize the impact of EIV infection. Emergency vaccination was implemented during the 2007 Australian EI outbreak as part of the control strategy, alongside movement restriction and disease surveillance. A live recombinant canarypox-based EI vaccine was selected, based on its ability to induce rapid onset of immunity, efficacy against contemporary EIV strains, successful use during the 2003 South African EI outbreak and DIVA characteristics. The last EI case was reported 5 months after the start of the outbreak. Responses to vaccination and other control measures have been rarely documented in horses with such details before. In our opinion, successful eradication of EI in Australia represents one of the greatest achievements of Veterinary Medicine in recent years.

## Abbreviations

The following abbreviations are used in this manuscript:

A$	Australian dollar
CCEAD	Consultative Committee for Emergency Animal Disease
DIVA	Differentiating Infected from Vaccinated Animals
DPI	Department of Primary Industries
EI	Equine Influenza
EIV	Equine Influenza Virus
ELISA	Enzyme-Linked ImmunoSorbent Assay
EU	European Union
FC1	Florida Clade 1
HA	Haemagglutinin
HI	Haemagglutination Inhibition
ISCOM	Immuno Stimulating Complex
MDPI	Multidisciplinary Digital Publishing Institute
NP	nucleoprotein
NSW	New South Wales
NZ	New Zealand
PAQ	Post-Arrival Quarantine
PEQ	Pre-Exportation Quarantine
PRISMA	Preferred reporting items for systematic reviews and meta-analyses
QLD	Queensland
qRT-PCR	quantitative Reverse Transcription Polymerase Chain Reaction
SRH	Single Radial Haemolysis
V	Vaccination (e.g., V1)
